# A Comparative Analysis of Oocyte Development in Mammals

**DOI:** 10.3390/cells9041002

**Published:** 2020-04-17

**Authors:** Rozenn Dalbies-Tran, Véronique Cadoret, Alice Desmarchais, Sébastien Elis, Virginie Maillard, Philippe Monget, Danielle Monniaux, Karine Reynaud, Marie Saint-Dizier, Svetlana Uzbekova

**Affiliations:** 1 INRAE, CNRS, Université de Tours, IFCE, PRC, F-37380 Nouzilly, France; 2 CHU Bretonneau, Médecine et Biologie de la Reproduction-CECOS, 37044 Tours, France

**Keywords:** oocyte, mammals, evolution, gene expression, posttranscriptional control, Gdf9, Bmp15, lipids

## Abstract

Sexual reproduction requires the fertilization of a female gamete after it has undergone optimal development. Various aspects of oocyte development and many molecular actors in this process are shared among mammals, but phylogeny and experimental data reveal species specificities. In this chapter, we will present these common and distinctive features with a focus on three points: the shaping of the oocyte transcriptome from evolutionarily conserved and rapidly evolving genes, the control of folliculogenesis and ovulation rate by oocyte-secreted Growth and Differentiation Factor 9 and Bone Morphogenetic Protein 15, and the importance of lipid metabolism.

## 1. Introduction

Sexual reproduction requires the fertilization of a female gamete after it has undergone optimal development. In animals, oogenesis has been studied in organisms as diverse as insects, worms, amphibians and mammals. In the latter, the mouse has long been the model of choice to delineate the complex mechanisms that regulate oogenesis and to identify the major genes and molecular actors involved throughout the process. However, in recent decades, progress in genomics has made it possible to investigate molecular mechanisms in an array of species, including farm animals. In this chapter, we discuss how various aspects of oocyte development and many molecular actors of this process are shared among mammals and possibly flies, birds and teleost fishes, but also how distinct mammalian species display specificities. We focus on gene expression in the oocyte, on the control of folliculogenesis and ovulation rate through the molecular dialog between the oocyte and granulosa cells, and finally, on the metabolism of oocyte lipids.

## 2. Gene Expression: Old Players and Newcomers Shape the Oocyte Transcriptome

### 2.1. Transcriptional Activity in the Oocyte Throughout Folliculogenesis

The oocyte displays an uncommon pattern of gene expression regulation, with partial uncoupling of transcription and translation. While some oocyte RNA are translated to ensure cellular metabolism, others are deadenylated and stored in the cytoplasm within ribonucleoparticles. An estimate of the RNA content of the fully grown oocyte in most mammalian species is 0.3–0.5 ng (mouse, human) to 0.7–2 ng (pig, sheep, cow), with the exception of rabbit, which has been reported to contain up to 15 ng [1,2,3,4,5,6]. This is much higher than in most if not all cells in the body, although it remains far below the microgram amount of RNA in chicken or *Xenopus* eggs.

As deduced from the nucleolus ultrastructure, from the localization of immunolabeled proteins involved in ribosomal RNA synthesis or maturation, and from incorporating radio-labelled uridine into elongating RNA, transcriptional activity in the oocyte appears discontinuous throughout folliculogenesis in various species (e.g., mouse and human [7,8], cow [9,10], pig [11]). Transcription is hardly detectable in the oocyte of primordial follicles. It is activated in the oocyte of primary follicles and becomes very intense during subsequent oocyte growth. It is progressively inactivated as the oocyte reaches its maximal diameter, in relation with species-specific rearrangements in large scale chromatin configurations that become more condensed in advanced oocytes [12].

Within this general pattern, differences are observed between species. In mouse, but also rat and human advanced oocytes, condensed chromatin forms a rim around the nucleolus, defining the surrounded nucleolus (SN) status. Beforehand, mouse oocyte undergoes a transition from non-SN to partly non-SN, then partly SN, and finally, SN [13]. A similar nomenclature is used in the pig, with a similar evolution of chromatin configuration through oocyte growth, except that chromatin eventually redecondenses in oocytes that will sustain embryo development; additional configurations of condensed NSN to condensed SN are observed, mostly in atretic follicles [14]. In the cow, chromatin evolves from a filamentous appearance in the whole germinal vesicle (GV0 status) of rapidly growing oocytes to displaying some small (GV1) then large (GV2) areas of condensed DNA, and forming a single large clump (GV3) during the final stages of oocyte growth [15]. In rabbits, a netlike condensed chromatin configuration is mainly observed in small growing oocytes; then, an increasing proportion of oocytes display a loosely condensed and tightly condensed chromatin configuration as follicles grow [16]. In the dog, similarly, as follicles grow, the proportion of oocytes with their chromatin homogeneously distributed throughout the nucleoplasm decreases, while the proportion of oocytes with their chromatin partly or highly condensed around the nucleolus increases. The latter configuration is most prevalent in ovulated oocytes, as ovulation occurs before meiotic maturation in this species [17,18].

Subsequent chromatin hypercondensation is the first sign of meiotic resumption before the germinal vesicle breaks down. Then, maturation proceeds in the absence of transcription. Following maturation and fertilization, transition from the maternal to the embryonic control of genome expression occurs progressively. Minor transcriptional activity has been detected early after fertilization, but transcription becomes intense only after one to several cell cycles, at a specific stage for each species: in 2-cell mouse embryos, 4/8-cell human and pig embryos, 8/16-cell ovine, bovine and rabbit embryos [19,20,21,22,23]. Overall, for one to several days, gene expression relies on factors stored in the oocyte and inherited by the embryo, and on posttranscriptional control of ribonucleoparticle-associated maternal transcripts.

### 2.2. Posttranscriptional Control of Maternal RNA

**Figure 1:**
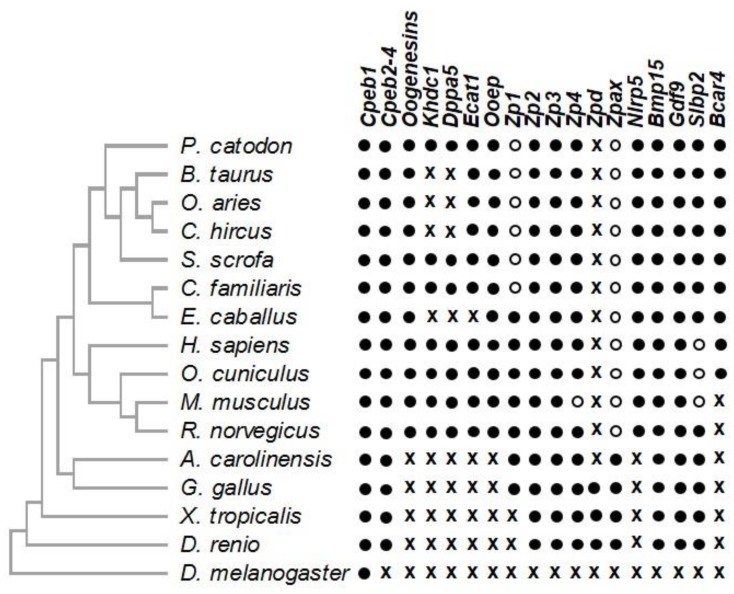
Evolution patterns of oocyte expressed genes in selected species. Colored and empty circles indicate the presence of a gene or pseudogene respectively; x indicate that no gene or pseudogene were reported or could be found. The species tree on the left is not to scale.

*Cis*-elements in the 3’ untranslated region (UTR) are involved in the storage of oocyte transcripts and in their recruitment for translational activation, which can occur through cytoplasmic polyadenylation-dependent or -independent mechanisms. In a complex combinatorial code, the sequence of the *cis* elements, their number and position relative to the 3’ end regulate the chronology of the recruitment, i.e., they orchestrate the timely recruitment of specific transcripts during early or late maturation or after fertilization [24,25]. Cytoplasmic polyadenylation is widely conserved in evolution, as it has been observed in eggs from *Drosophila*, *Xenopus* and fishes, as well as in mammalian oocytes. The molecular mechanisms were mainly elucidated in *Xenopus* (reviewed in [26]). A U-rich cytoplasmic polyadenylation element (CPE) in the 3’ UTR can recruit a CPE-binding protein (CPEB) in a complex including the Cleavage and Polyadenylation Specificity Factor (CPSF) and the Terminal Nucleotidyltransferase 2 which catalyzes polyA tail elongation. Cytoplasmic polyadenylation has also been studied in the mouse (reviewed in [27]), while sparse data are available in the cow [28,29,30,31] and the pig [32,33]. Owing to the limited availability of information, a detailed comparison between mammalian species is premature. We will focus on the central factor CPEB. Four *CPEB* genes are conserved in mammals [34], in the chicken, in the zebrafish and in *Xenopus* (Ensembl gene trees ENSGT00940000155524, ENSGT00940000160357, ENSGT00940000158949, ENSGT00940000154998 for *CPEB1-4* respectively), with *CPEB1* being reported as the ortholog to the *Drosophila Orb* gene (Figure 1). Distinct CPEB proteins might function in the posttranscriptional control of maternal RNA in distinct mammalian species, as the relative abundance of the four CPEB transcripts varies between mammalian species, i.e., CPEB1 is the most abundant in the mouse and human immature oocytes, while CPEB4 is the most abundant in the bovine immature oocyte ([30] and personal data). In bovine oocyte, CPEB1 phosphorylation and degradation accompany the successive steps of oocyte meiotic maturation [35]. In pig oocytes, inhibiting CPEB1 phosphorylation or knock-downing CPEB2 alters oocyte nuclear maturation [32,33], suggesting that they are both involved. It is also important to mention that CPEB are involved in oogenesis long before meiotic maturation, as shown by the arrest of meiosis at pachytene stage or abnormal oocyte and follicle growth in mice when CPEB1 is inactivated initially or after follicle formation [36,37]. It is possible that several CPEB are involved successively throughout oocyte growth, maturation and after fertilization, as suggested in *Xenopus* [26].

### 2.3. The Oocyte Transcriptome

Transcriptomics has revealed the profile of oocyte RNA, including “oocyte specific genes” that are transcribed only or strongly preferentially in the female gamete, as compared to somatic or male germ cells. Distinct patterns of evolutionary history are deduced from molecular phylogenetic analysis.

#### 2.3.1. Genes Highly Conserved in Fly or Distant Vertebrates and the Mouse

A first group of genes are conserved from *Drosophila* to mammals. We searched for the mammalian orthologs of some 971 genes known to be involved in oogenesis in *Drosophila*. This meta-analysis revealed a remarkably high proportion of conservation, as 78% of these genes have at least one ortholog in the mouse, as compared to 42% of all genes in the fly genome. Approximately 15% of these mouse orthologs are expressed exclusively or most preferentially in the oocyte as compared to other cells [38]. Beyond meiosis, several functions such as mRNA binding seem to be highly conserved in oocytes from nonvertebrates to vertebrates [38,39]. This is not unexpected, given the prominent role of posttranscriptional control of gene expression, as seen in §2.2. The *Karyopherin* (*Kpn*) genes encodes importins involved in nucleo-cytoplasmic transport. Kpna7 and Kpna2 transcripts are very abundant in fully-grown oocytes of the mouse [40] and the cow (personal communication). KPNA7 interacts with other oocyte-preferential factors such as Nucleoplasmin2 in bovine and Stl3 in trout [41,42]. It is required for optimal preimplantation development of mouse, bovine and pig embryos [41,43,44].

Several studies have revealed a high conservation of genes specifically expressed in oocytes between distant vertebrates, in particular between the frog, the cow and the mouse [45,46]. These genes encode proteins involved in particular in RNA binding and metabolism, as well as in early embryonic development. Among them, some genes seem to have appeared in vertebrate species such as *bone morphogenetic protein 15* (*BMP15*) and *growth differentiation factor 9* (*GDF9*) (Figure 1) that are crucial for the dialogue between the oocyte and the granulosa cells from primary to preovulatory follicles (see below 3.3).

#### 2.3.2. Genes That Appeared Specifically in Mammals

By screening databases of cDNA libraries from murine oocytes versus somatic (nonpathologic) tissues, we and others have identified novel genes which are preferentially, if not exclusively, expressed in the oocyte. To validate this bioinformatic methodology, the specificity of expression was confirmed experimentally by RT-PCR in a range of organs and/or by in situ hybridization onto ovarian cross-sections [47,48,49,50,51].

**Figure 2:**
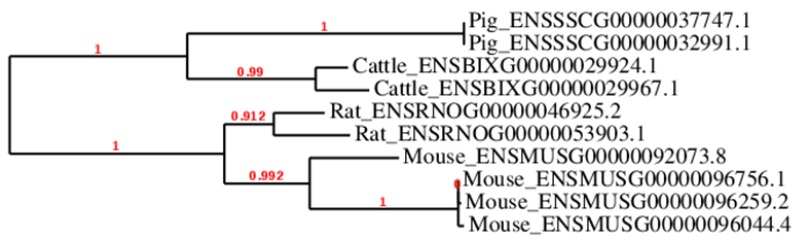
Simplified tree of selected mouse, rat, bovine and porcine oogenesins illustrating the gene duplications that took place after speciation or divergence of these species. Branch lengths correspond to evolutionary distance. Bootstrap values in red indicate the confidence of the node.

Some of these genes belong to families of paralogs whose many members are preferentially expressed in oocytes: *Oogenesins*, *Nlrp*, *Khdc1/Dppa5/Ecat1/Ooep*. Among them, *KHDC1* and *DPPA5* (gene tree ENSGT00940000154353) are absent from the genomes of several mammals including ruminants and horse (Figure 1). *Nucleotide-binding oligomerization domain, Leucine Rich repeat and Pyrin domain containing 9* (*NLRP9*), one of the bovine paralogs of *NLRP5* (also known as *MATER*), is not only present in the genome, but is also specifically expressed in the oocyte of the cow [52]. Interestingly, from an evolutionary point of view, many of these genes specifically appeared in eutherians, and have evolved very rapidly. Some even appear to have duplicated after divergence from the nearest species (Figure 2), as if each species had its own set of oocyte genes, and putatively its own strategy for some oocyte biological functions. Interestingly, from a functional point of view, although the *Nlrp* paralog genes encode very similar proteins, the knock-out of *Nlrp5* alone leads to the arrest of embryonic development at the 2-cell stage; this is not compensated for by the presence of other paralogs, suggesting that there is no functional redundancy [53]. Similarly, knockdown of *Nlrp14* is sufficient to alter embryo development [54]. It would be interesting to test if the absence of functional redundancy also concerns the oogenesin family [48], the *Khdc1/Dppa5/Ecat1/Ooep* family [51] and the other families of oocyte specific genes that we have identified so far [55].

Other genes have been identified only in a subset of mammalian species, excluding other taxa. Although it cannot be excluded that high sequence divergence might prevent identifying them in some species, they most likely appeared and/or were lost after speciation events within this taxon. *Breast Cancer Anti-estrogen Resistance 4* (*BCAR4*) was discovered in parallel as an oocyte-specific gene in bovine [56,57] with ectopic expression in a subset of human breast tumors [58]. Our investigation uncovered orthologs in rabbit [59], sheep, dog, horse, chimpanzee, orangutan and macaque [57] as well as in goat (ENSCHIG00000005110), sperm whale (ENSPCTG00005010916), and most primates, although it often remains unannotated (Figure 1). The gene displays a remarkable sequence divergence, with only 30% identity between the human and bovine putative protein sequences, corresponding, for the most part, to two predicted transmembrane domains. A gene or pseudogene could not be found in the syntenic region of the mouse and rat genomes. In these two species, embryonic genome activation (EGA) occurs as early as one cell-cycle after fertilization. BCAR4-targeting small interfering RNA injected into bovine oocytes reduces the rate of blastocyst formation in vitro [57]. We suggest that BCAR4 is involved in cell division in the pre-EGA embryo, consistent with its proproliferative activity in various cancers.

#### 2.3.3. Genes Lost in Several Vertebrate Species

##### Genes Involved in Controlling Histone Translation

Some oocyte-expressed genes can be conserved in distant vertebrates but may have been lost in several species. The Stem-Loop Binding Protein (SLBP) interacts with the evolutionarily conserved stem-loop structure in the 3’ untranslated region of most histone mRNA, to control RNA stability and translation. It is conserved in virtually all vertebrates and displays low tissue specificity. An oocyte-specific paralog *SLBP2* was first characterized in *Xenopus* [60]. In stage-VI oocytes, SLBP2 binds to and protects histone mRNA from premature translation. During maturation, it is degraded and replaced with SLBP to ensure histone synthesis. The first mammalian *SLBP2* ortholog was characterized in the cow [61,62]. Bovine SLBP2 protein was detected in both immature fully grown and mature oocytes, and in pre-EGA embryos. Based on Ensembl gene tree ENSGT00940000164705, a protein-coding gene was identified in the vast majority of Laurasiatheria, in three primates (marmoset and two old world monkeys) and seven rodents including rat. By contrast, a pseudogene was found in the human, mouse and rabbit genomes (Figure 1). This evolutive pattern suggests that the control of histone translation in the oocyte and early embryo relies on distinct mechanisms, depending on the species.

##### Genes Involved in Fertilization

Genes encoding the proteins that compose the Zona Pellucida (ZP) are another example. There are almost ten *ZP* genes in teleost genomes, six in the chicken genome (*ZP1, ZP2, ZP3, ZP4, ZPD and ZPAX*), four in the human and the rat genomes (*ZP1, ZP2, ZP3* and *ZP4*), three in the cow and the dog genome (*ZP1, ZP2 and ZP4*), and three as well in the mouse genome (*Zp1, Zp2 and Zp3*) (Figure 1). Combining sequence alignment and the analysis of synteny allowed us to demonstrate that the *ZPAX* gene was lost by pseudogenization in the human genome, as were *Zp4* in the mouse and *ZP1* in the dog, suggesting that several genes encoding ZP proteins have been progressively and systematically lost during the evolution of vertebrates [63].

We extended the study to the 69 genes encoding proteins known experimentally to be involved in the three stages of fertilization: the sperm/egg interaction, the acrosomal reaction and the membrane fusion. The genes involved in the first stage tend to be lost in mammals, and those involved in membrane fusion tend to duplicate and evolve under positive selection in bony fishes [64]. The loss of gene encoding proteins involved in sperm/egg interaction is likely related to the mode of internal and external fertilization, respectively in mammals and teleost. It can be hypothesized that the corresponding genes become relaxed in mammals in which other species barriers exist (sexual behavior and the impossibility of encountering gametes of two different mammals), whereas a very strict physical barrier is important in externally fertilized species, for which gametes of different species or genera are likely to mix.

## 3. The Oocyte is a Driver of Folliculogenesis and Can Control the Ovulation Rate

### 3.1. The Oocyte Drives Follicular Cell Proliferation and Differentiation

The existence of a privileged dialog between the oocyte and its surrounding follicular cells coordinating the different phases of follicular development is now well established (for review: [65,66,67,68,69]). Since the discovery of an antiluteinizing role of the oocyte in the rabbit [70], the central role of the oocyte on its companion somatic cells has been established by numerous studies in various animal species.

In rodents, the oocyte has been shown to maintain the granulosa cell lineage phenotype [71]. Moreover, the complete elimination of growing oocytes by oocyte-specific inactivation of *Omcg1* (encoding a nuclear zinc finger protein involved in pre-mRNA processing) induces dramatic structural and functional changes of the ovarian somatic compartment, allowing the interstitial tissue to produce oestradiol [72].

During follicular growth, the oocyte promotes the proliferation of granulosa cells (mouse: [73]; rat and cow: [74]; cow: [75]) and orchestrates the rate of follicular development (mouse: [76]). In humans, the polycystic ovarian syndrome (PCOS) is associated with a dysregulated dialog between oocyte and granulosa cells, leading to an increased granulosa cell proliferation in early-growing follicles [77].

Moreover, in antral follicles, the oocyte inhibits the differentiation of cumulus cells into mural granulosa cells, particularly through the inhibition of LH receptor expression (mouse: [78] and progesterone production in mouse, rat, pig and cow cumulus cells [75,79,80,81,82]. In the mouse preovulatory follicle, the oocyte has been shown to promote cumulus expansion [83] by stimulating the synthesis of hyaluronic acid [84]. More recently, it was shown that the mouse oocyte promotes the expression of *Npr2* in cumulus cells (encoding the receptor of the natriuretic peptide precursor type C), thus participating in meiotic arrest [85].

### 3.2. The Underlying Mechanisms

From our current knowledge, the dialog between the oocyte and its surrounding granulosa/cumulus cells operates through two types of mechanisms.

The first mechanism is based on molecular exchanges through intercellular channels consisting of gap junctions located at the tips of granulosa/cumulus cell transzonal cytoplasmic projections. In human growing follicles, long and thin follicular ‘‘intra-ooplasmic processes’’ have been seen to penetrate deeply into some oolemma invaginations, coming very close to the nucleus and making contact with different oocyte organelles [86]. In the mouse, the oocyte itself was recently shown to induce the formation of these processes, identified as filopodia [87]. In fully-grown oocytes, cAMP and cGMP, both originating in granulosa cells and transferred to the oocyte via gap junctions, are crucial for maintaining meiotic arrest [88,89].

The canine oocyte constitutes a unique model of meiosis resumption among mammals. At ovulation, dog oocytes are released at the germinal vesicle (GV) stage and resume meiosis 2–3 days later in the oviduct [90]. In contrast with other mammals, cumulus cells remain firmly attached to the dog oocyte zona pellucida for 2–3 days after ovulation [17]. Transzonal projections from the granulosa cells to the oocyte cytoplasm are also maintained for 2 days after ovulation, and retract in parallel with meiosis resumption at 3 days postovulation [91]. However, cAMP does not seem to have a central role in dog oocyte meiosis resumption; the addition of protein kinase A or dibutyryl cAMP, a stable cAMP analogue, in the culture medium did not have any effect on in vitro maturation of canine oocytes [90,92]. We do not further discuss this mechanism of oocyte meiosis regulation in this review.

The second mechanism is based on the production, by one cell type, of cytokines and growth factors that we have mentioned above, which can bind specific receptors present on the other cell type and activate signaling pathways. Particularly, two landmark studies demonstrated that the absence of two oocyte-specific growth factors, GDF9 and BMP15, causes sterility (mouse: [93]; sheep: [94]). Further studies have demonstrated that these molecules are central regulators of granulosa/cumulus differentiation and may be associated with the pathogenesis of ovarian dysfunction (for review: [67,95,96]. This aspect is developed below, and comparisons between species are discussed in this review.

### 3.3. The Oocyte-Secreted Factors GDF9 and BMP15 Regulate Granulosa and Cumulus Cell Function

#### 3.3.1. Structural Features of GDF9 and BMP15 and Common Roles

As with other members of the transforming growth factor beta family, *GDF9* and *BMP15* are transcribed to encode preproproteins comprised of a signal peptide, a large proregion and a mature region, which lacks the cysteine residue normally involved in the formation of a covalent dimer [97,98,99]. Both GDF9 and BMP15 can form noncovalent homodimers when expressed individually, while when both are coexpressed then GDF9/BMP15 heterodimers are produced [100]. Interestingly, GDF9 and BMP15 are preferentially expressed in the same cell type, i.e., the oocyte, as shown in the mouse, the rat, the cow, the sheep, the pig and the human [98,99,101,102,103,104,105,106,107]. In the mouse, the presence of the GDF9 protein has also been detected in the expanded cumulus and the follicular fluid [108]. Apart from rodents, GDF9 and BMP15 may also be expressed, although at a much lower level, by cumulus cells in the pig [109], the goat [110], the human [111], the cow [112] and the dog [113,114].

In all mammals, GDF9 and BMP15 can act alone or cooperate, acting in synergy or as heterodimers on granulosa/cumulus cells. Mouse and human GDF9/BMP15 heterodimers are the most bioactive ligands on mouse granulosa cells compared with homodimers [115]. The generation of human recombinant heterodimeric complexes of promature domains (named procumulin) and covalent mature domains (named cumulin) showed that both procumulin and cumulin exhibit highly potent bioactivity on granulosa cells, activating both SMAD2/3 and SMAD1/5/8 signaling pathways, and promoting the proliferation and expression of a set of genes associated with oocyte-regulated granulosa cells [116].

In relation with the close structural similarity of GDF9 and BMP15 and their ability to cooperate, they can exert similar effects when acting on granulosa/cumulus cells. Indeed, GDF9 inhibits FSH-stimulated progesterone production by granulosa cells, preventing their premature luteinization in rodents [106,117], pigs [118] and cows [119]; a similar effect has been demonstrated for BMP15 acting on rat [106], sheep [120] and human [121] granulosa cells. In the mouse, GDF9 and BMP15 cooperate to enhance the growth of preantral follicles in vitro [122]. In vivo, since the mature form is not detected at this stage, this likely involves the BMP15 proprotein, whose proregion is able to induce the formation of a heterodimer with GDF9 [123]. Both BMP15 and GDF9 also promote cholesterol biosynthesis in cumulus cells of antral follicles, and are required for EGF receptor expression in these cells [124]. BMP15 and GDF9 coordinate with estradiol to promote the development of cumulus cells and maintain their competence to undergo expansion [125]. The GDF9/BMP15 heterodimer also promotes the expression of *Impdh* and *Npr2* and elevates cGMP levels in cumulus cells. Thus, the maintenance of oocyte meiotic arrest is regulated by signals from the oocyte itself [126].

As stated earlier, the canine oocyte displays very uncommon features of meiosis resumption. In this species, the expression of GDF9 and BMP15 proteins has been reported to decline in follicular cells with increasing follicle size during estrus [113], possibly in a species-specific manner related to the delay in cumulus expansion after ovulation. Furthermore, GDF9 was reported at high levels only in immature GV oocytes, but was barely detectable in metaphase II oocytes after in vitro maturation [127]. GDF9 and BMP15 may cooperate in the process of oocyte meiosis resumption in dogs. Supplementing in vitro cultures with both recombinant human proteins (supplied as homodimers of the mature region) increases the rates of oocytes reaching metaphase II, while this improvement is not observed with either GDF9 or BMP15 added individually [128]. However, the pathways by which GDF9 and BMP15 may interact synergistically to improve oocyte meiosis competence is currently unknown.

In the human, different mutations in both *GDF9* and *BMP15* contribute to premature ovarian insufficiency (POI, premature menopause) [129,130,131,132,133,134,135,136,137]. The first substitution mutation identified in humans with hypergonadotropic ovarian failure is within the proregion of the BMP15 proprotein (Y235C) [129]. Most mutations associated with POI have been found in the proregions of BMP15 and GDF9 (for review: [138]). Interestingly, three prodomain mutations associated with POI (S186Y, V216M, and T238A) result in the activation of human GDF9. Mechanistically, these mutations reduce the affinity of the prodomain for mature GDF9, allowing the growth factor to more readily access its signaling receptors [139]. Based on the potent mitogenic activity of GDF9 onto granulosa cells [140], increased activity of GDF9 in these women may increase the proportion of growing follicles, leading to a premature depletion of the ovarian reserve. However, it seems that there is no systematic correlation between the genotype and the phenotype among GDF9 mutations (i.e., mutations that either decrease expression or activate human GDF9 are associated with POI).

#### 3.3.2. Specific Roles of GDF9 and BMP15

Despite their strong structural similarities and their ability to interact and cooperate, GDF9 and BMP15 do not seem to play an exactly identical role during follicular development. For instance, GDF9 inhibits in vitro the expression of the cytokine *Kitlg* in mouse granulosa cells, whereas BMP15 promotes *Kitlg* expression in monolayers of granulosa cells from rat early antral follicles [141]. It is increasingly evident that GDF9 and BMP15 exert specific effects on their surrounding somatic cells.

Various specific effects of GDF9 have been reported. In the mouse, the invalidation by transgenesis of *Gdf9* leads to the loss of transzonal projections [142], whereas GDF9 treatment of granulosa cells induces the formation of new transzonal projections during oocyte growth [87]. Moreover, GDF9 stimulates the production of Dhh/Ihh by the granulosa cells of small growing follicles, thereby inducing the recruitment of theca cells from interstitial ovarian cells [143]. In antral follicles, GDF9 inhibits *Lhcgr* expression and stimulates *Has2* and *Ptgs2* expression in granulosa/cumulus cells [144,145]. Moreover, GDF9 maintains the integrity of the cumulus-oocyte complex in preovulatory follicles and after ovulation [146], particularly by promoting the synthesis of PTX3 [147]. In the human, GDF9 has been shown to promote the development of primordial follicles to the secondary stage in culture, as well as to improve follicular survival [148]. A decrease in *GDF9* mRNA has been reported in polycystic ovary disease, a common cause of female infertility [149].

Some specific effects of BMP15 have also been reported. In rodents, BMP15 inhibits the expression of the FSH receptor in granulosa cells [150] and can induce the atresia of preantral follicles in vitro [122]. In antral follicles, BMP15 has been shown to control the expression of genes involved in cumulus metabolism (mouse: [151]; cow: [152]) and expansion (mouse: [153]), and to increase oocyte developmental competence (cow: [154]). Cooperation between BMP15 and the oocyte-secreted fibroblast growth factor 8 (FGF8) has been shown to regulate cumulus cell glycolysis in the mouse [155]. In the cow, BMP15 and FGF10 cooperate to stimulate the expansion of in vitro-matured cumulus-oocyte complexes by driving glucose metabolism toward hyaluronic acid production and controlling the expression of genes in the ovulatory cascade, with the former acting upon *ADAM10*, *ADAM17*, *AREG* and *EREG* and the latter on downstream genes, particularly *PTGS2* [152].

**Figure 3:**
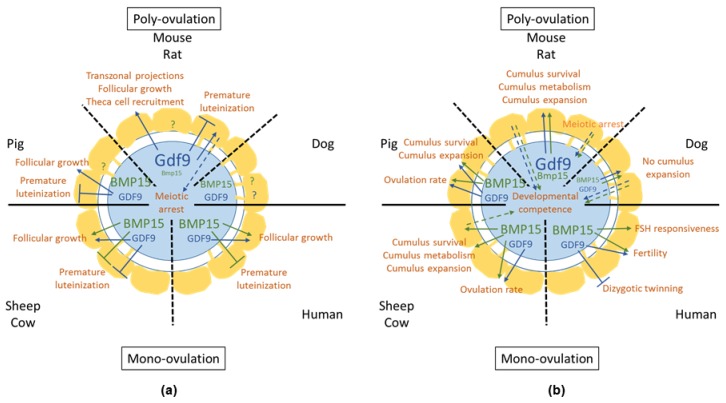
Comparison of the role of oocyte-derived BMP15 and GDF9 (**a**) in small growing follicles and (**b**) in large antral and preovulatory follicles. For *GDF9* and *BMP15*, letter size symbolizes the expression level. Solid lines and arrows indicate the effect of GDF9 (blue) and BMP15 (green) onto somatic cells (and onto follicular development and ovulation). Dotted lines and arrows indicate feedback actions of somatic cells onto the oocyte.

Interestingly, the role of BMP15 in ovarian function can differ between mammals (Figure 3). Indeed, both GDF9 and BMP15, acting in vitro alone and in synergy, enhance granulosa cell proliferation in rodents [106,117,156,157], but GDF9 only has been shown to exert a proliferative effect on pig [118] and cow [119] granulosa cells. Moreover, the mature form of BMP15 is not expressed before the preovulatory follicle stage in the mouse [158], unlike nonrodent species, in which it is expressed from the primary follicle stage onwards. Furthermore, genetic models have shown striking differences between species for the role of BMP15 in ovarian function. Indeed, the invalidation by transgenesis of *Bmp15* in the mouse does not impair follicular development and ovulation, and the *Bmp15-/-* mice are fertile [146], whereas sheep carrying homozygous inactivating *BMP15* natural mutations are sterile, due to the arrest of folliculogenesis at the primary follicle sage [94,159,160,161]. In humans, the genotypes *CT* and *TT* from *BMP15:c.852C>T* variation are risk factors for the development of POI [162]. Unlike *BMP15* mutant sheep and humans carrying a *BMP15* mutation, the lack of bioactive BMP15 does not prevent follicles from progressing through the component stages of folliculogenesis in mice.

### 3.4. Can Oocyte-Secreted Factors GDF9 and BMP15 Control the Ovulation Rate?

Whereas ewes carrying homozygous inactivating natural mutations of the *GDF9* gene are sterile, the presence of *FecG^H^*, or *FecG^T^* mutations at the heterozygous state in the *GDF9* gene leads to hyperprolificacy, due to increased ovulation rates in carrier ewes [163,164]. As an exception, the presence of the *FecG^E^* mutation in the *GDF9* gene is associated with hyperprolificacy of the heterozygous, and even more the homozygous ewes [165].

In a similar way, ewes carrying the homozygous inactivating natural mutations *FecX^I^*, *FecX^H^*, *FecX^B^*, *FecX^G^*, *FecX^L^*, or *FecX^R^* of the *BMP15* gene are sterile, whereas all heterozygous ewes are hyperprolific [94,161,163,166,167]. The reduction in bioactive BMP15 that would be expected in these animals would lead to an increase in the sensitivity of follicles to FSH, an accelerated follicle development and precocious ovulation of small follicles [120,168]. Sheep that are heterozygous carriers of the partially inactivating *FecBB* mutation in the *ALK6* gene (encoding the BMPR1B receptor of the BMP15/BMP15 homodimer and the BMP15/GDF9 heterodimer) also exhibit an increased ovulation rate, similar to the phenotype of heterozygous mutant *BMP15* ewes [169,170,171]. Interestingly, ewes which are homozygous carriers of the *FecBB* mutation exhibit ovulation rates which are even higher than those of the heterozygous carriers of the mutation. Recently, the presence of the *FecXG^R^* or *FecX^O^* mutations in the *BMP15* gene has been found to be associated with hyperprolificacy of the heterozygous and even more the homozygous ewes [172].

The immunization approaches led by the group of Juengel in New Zealand demonstrated the possibility that BMP15 and GDF9 could represent therapeutic targets to control fertility. Indeed, the effect on ovulation rate and sterility can be reproduced in sheep and cattle through adapted immunization regimens, neutralizing more or less BMP15 or GDF9, and confirms the crucial role of these two factors [173,174,175,176].

Overall, these observations in the sheep suggest that lowering the amounts of BMP15 or GDF9, or of the BMP15/BMP15 or BMP15/GDF9 signaling levels, are associated with the presence of multiple ovulations; conversely, mono-ovulating females would have higher levels of these factors. This assumption might also be true when considering mutations in other animal species (Figure 3). Indeed, low ovulation rates and prolificacy in Piau gilts have been shown to be associated with a greater abundance of genes controlling oocyte-secreted factors (*GDF9*, *BMP15* and *BMP6*) [177]. Recently, the overexpression of *SMAD6*, an inhibitor of the BMP/SMAD signaling, has been associated with a high ovulation rate in cows [178]. In the human, *GDF9* mutations are associated with dizygotic twinning [179,180]. Mutations observed in mothers of dizygotic twins (P103S and P374L) completely abrogate *GDF9* expression, suggesting that women heterozygous for these mutations would have a 50% reduction in GDF9 levels [139]. There is also some evidence that certain polymorphisms in the *BMP15* gene can predispose women undergoing fertility treatment to overrespond to FSH [181].

However, comparisons between species with different natural ovulation rates do not fully support this assumption. Indeed, high expression levels of *BMP15* have been shown in both mono-ovulating animals, such as ruminant species, and the highly polyovulating porcine species [182]. In the mouse, a polyovulating species, low levels of mature BMP15, but high levels of GDF9, are expressed [158], suggesting that the ratio GDF9:BMP15 might be important to regulate the ovulation rate. It has been shown that rat, pig, sheep, and red deer oocytes express species-specific ratios of *GDF9:BMP15* mRNA; with the exception of the pig, these ratios are directly correlated to litter size [182]. The granulosa cells of each species would have evolved to respond to these unique ratios [183].

## 4. The Oocyte: A High Energy Demanding Cell that Can Sense Changes in Its Lipid Environment

Nutritional changes affecting the energy balance or specific nutrients can affect folliculogenesis and ovulation by acting on the hypothalamo-pituitary system and/or directly on the ovary. Indeed, high body mass index (BMI) is related to an earlier puberty. Overweight women also exhibit a higher frequency of anovulation, infertility, poorer oocyte quality and maturity, and negative outcomes for obese patients undergoing in vitro fertilization (IVF) (reviewed in [184,185]). Increased BMI is also associated with quantitative alteration of endocrine factors, i.e., increased insulin resistance and hyperinsulinemia [186]. These alterations can adversely affect folliculogenesis and oocyte maturation by altering the expression of the genes involved in meiosis and steroidogenic pathways [187,188,189]. The metabolic sensors mediating these effects will not be detailed in this section (adipokines, AMPK, peroxisome proliferator-activated receptor (PPAR) etc.) as they have been well reviewed elsewhere [190,191,192]. This section will focus on nutrition and metabolic changes, especially lipid metabolism, affecting oocyte quality and differences occurring between mammal species.

### 4.1. Primary Nutrients Needed for Oocyte Development

The oocyte is physically and metabolically coupled with surrounding granulosa or cumulus cells, and is able to use several energy substrates such as carbohydrates and amino acids, but also lipids.

#### 4.1.1. Carbohydrates

Pyruvate is the main energy substrate oxidized in follicles, where both glycolysis and mitochondrial pyruvate oxidation occur, and pyruvate is required to meet oocyte energetic requirement for the resumption of meiosis (reviewed by [193,194]). Moreover, dietary glycemic load (quality/amount of carbohydrate) is related to anovulation [195]. In the ovaries, glucose is essential for regulating meiotic maturation and determining oocyte developmental competence [191,196].

#### 4.1.2. Amino Acids and Proteins

Some data have reported the importance of amino acids on the oocyte, such as their effects on ovulation or oocyte quality (reviewed by [193]). The oocyte amino acid transport and composition are related to the oocyte maturation stage and the ability of oocyte to cleave once fertilized (reviewed by [193]). In the cow, glycine and alanine from follicular fluid were even suggested as predictors of cumulus-oocyte complex quality [197]. Finally, amino acid depletion impairs mechanistic Target Of Rapamycin (mTORC) signaling, and consequently disturbs folliculogenesis and oogenesis (reviewed in [198]).

#### 4.1.3. Lipids

More and more data point out the role of lipids in female fertility. Indeed, oocyte lipid metabolism is one of the main functions modulated by the absence of cumulus cells surrounding the bovine oocyte and that affects its maturation rate [199]. Lipid metabolism is among the significantly affected biological functions when comparing the transcriptome of ewe follicles at different stages of early folliculogenesis [200]. Moreover, fatty acid β-oxidation in cumulus cells is essential for oocyte maturation in different mammalian species, such as the mouse, cow and sow [201,202,203]. In humans, a high level of free fatty acids in follicular fluid (as observed in obese patients) will lead to lipo-toxicity and inhibit oocyte maturation. Exposure of bovine oocytes to a high concentration of nonesterified fatty acids during in vitro maturation can impair maturation itself, as well as subsequent fertilization and embryo development. Additionally, the resulting blastocysts display an altered pattern of gene expression and epigenetic modifications [204,205]. Cumulus cells indeed incorporate lipids from follicular fluid and store them in lipid droplets to protect the oocyte from lipo-toxicity (reviewed by [184]).

Saturated fatty acid supplementation during in vitro maturation reduces bovine blastocyst rate, suggesting a reduction in oocyte quality. It also modulates the expression of genes related to apoptosis, oxidative stress and oxidative metabolism (increase in *GPX1* and *GAPDH* expression in the bovine oocyte, reduction in *GAPDH*, *GPX1*, *G6PD* and *LDHA* in cumulus cells [206]).

In contrast, dietary fat supply, especially with polyunsaturated fatty acids (PUFA), has been used to improve cow fertility (reviewed in [207]). Supplementation with PUFA during oocyte maturation in vitro does not impair, and could even enhance, bovine oocyte quality [206,208]. A PUFA, namely oleic acid, could restore the quality of bovine oocyte that had been previously exposed to high levels of saturated fatty acids, partly through an increase in the amount of lipids stored in lipid droplets of cumulus cells [209]. In bovine, n-3 PUFA supplementation increases gestation rate and the cryotolerance of in vitro produced embryos, and reduces early embryo mortality in vivo [209,210,211]. Such dietary supplementation has also been reported to potentially enhance oocyte quality in the ewe [212], whereas it can be detrimental in the mouse [213]. Complementary in vitro experiments suggested that part of these n-3 PUFA beneficial effects occurs through direct effects on the oocyte, as evidenced by blastocyst transition rates after in vitro fertilization. Indeed, n-3 PUFA alpha linolenic acid is able to restore bovine oocyte quality under high lipotoxic fatty acid conditions, notably by protecting cumulus cell viability [208]. Moreover, supplementing in vitro maturation media with n-3 PUFA docosahexaenoic acid improves bovine oocyte quality and increases the blastocyst rate and blastocyst cell number, partly through the activation of Free Fatty Acid Receptor 4 [214,215]. In swine, supplementation with n-3 PUFA docosahexaenoic acid leads to increased cleavage rates and embryo cell numbers [216].

### 4.2. Lipid Metabolism

#### 4.2.1. Oocyte Lipid Composition

Oocytes contain abundant transcripts from genes involved in lipid metabolism and exhibit lipid droplets. Lipid droplets are an energy reserve of neutral lipids and composed of choline esters, triacylglycerols and proteins [217]. Lipids accumulated in the oocytes and in cumulus cells are used throughout oocyte maturation and early embryo development. Fatty acid oxidation is an essential source of energy for the oocyte. Indeed, the expression of numerous genes involved in fatty acid oxidation is regulated by LH peak surge, during oocyte maturation [202].

Moreover, analyses of lipids performed by matrix-assisted laser desorption/ionization time-of-flight mass spectrometry (MALDI-TOF MS) imaging on porcine ovarian sections reported the spatial distribution of more than 150 lipid species between ovarian follicular compartments. Lipid profiling was able to discriminate the theca, the granulosa cells, the follicular fluid and the cumulus-oocyte complex [218]. A similar methodology, using liquid chromatography and mass spectrometry, was performed on the bovine ovary. From the MALDI-TOF MS lipid fingerprints of follicular fluid, oocyte, theca, granulosa and cumulus cells, and the MS imaging of ovarian sections, the most abundant lipids in each compartment were identified and specific lipid contents were described for each compartment [217].

To further analyze lipid metabolism in the ovary, complementary transcriptional studies of the genes involved in lipid metabolism were performed. In porcine oocytes, an overexpression of *Cluster determinant 36* (*CD36)* (involved in lipid transport), *acetyl-coenzyme-A carboxylase* (*ACACA*), and *Perilipin 2* (*Plin2)* (involved in fatty acid storage in lipid droplets) as compared to theca, granulosa and cumulus cells was reported [218]. In contrast, lower expression of *Carnitine Palmitoyl Transferase 1a* (*CPT1a*) (involved in fatty acid oxidation and in ATP production) was evidenced in the oocyte compared to theca and granulosa cells. In bovine follicular cells, transcriptomic analyses confirmed differences in fatty acid metabolism-related genes and genes involved in steroidogenesis between the different ovarian follicular compartments, suggesting a crucial role of different lipids in the maintenance of energy homeostasis in the follicle [217]. Moreover, in the cow, the lipid content, analyzed by Desorption Electrospray Ionization Mass Spectrometry, was sufficient to discriminate the in vivo mature oocyte from the in vitro matured oocyte [219]. This was supported by differences in expression of genes involved in lipid metabolism (*Acetyl-CoA Acetyltransferase 1* (*ACAT1*), *Fatty Acid* Synthase (*FASN*) and *SREBP Cleavage-Activating Protein* (*SCAP*)).

The number of lipid droplets in the oocyte can correlate with its cytoplasmic maturation status and, therefore, with its competence to support embryo development [199,220]. Moreover, a dietary supplementation in n-3 PUFA, as mentioned in the previous paragraph, is able to modulate the lipid composition of bovine oocytes [210]. Such modifications of oocyte lipid composition are also reported after in vitro supplementation of n-3 PUFA during maturation in the bovine [215] or of conjugated linoleic acid in the porcine [221].

The bovine cumulus-oocyte complex is able to direct toward the desaturation of fatty acids through the stearoyl-CoA desaturase (SCD) enzyme [221]. Such changes consequently reduce the amount of saturated fatty acids, and therefore, protect the oocyte against lipotoxicity. In addition to the passive role of the oocyte in accumulating lipids from its environment into lipid droplets, some recent data suggest that the oocyte could also play an active role in modifying the stored lipids. Indeed, several genes of the *ELOngation of Very Long chain fatty acids protein* (*ELOVL)* family, that are able to build long chain fatty acids from shorter precursors, are expressed at a higher level in the bovine oocyte than in follicular somatic cells [217]; they are *ELVOL3*, *ELOVL4*, *ELOVL5* and *ELOVL7*. In ewe follicles, the expression of *ELOVL2*, *ELOVL4*, *ELOVL5* and *ELOVL6* was also reported through different stages of folliculogenesis in the oocytes ([200] and personal unpublished data).

#### 4.2.2. Lipid Requirement for Oocyte and Follicular Cells Functions

Alterations in energy metabolism, and therefore, in ATP production, lead to alterations in oocyte maturation. Indeed, pharmacological alteration of fatty acid oxidation (using etomoxir, C75, malonyl-CoA, mercaptoacetate) causes an arrest of the meiosis resumption induced by FSH or an AMPK activator in mice [222,223]. In the cow, oocyte maturation, as well as granulosa cell proliferation and progesterone secretion, are inhibited by etomoxir and mildronate, two inhibitors of fatty acid oxidation [203,224]. It is also possible to affect fatty acid oxidation by using peroxisome proliferator-activated receptor (PPAR) agonists. This subsequently leads to a decrease of embryo quality in the mouse [202]. In contrast, stimulating fatty acid oxidation through supplementation of the maturation medium with L-carnitine, the cofactor enabling the transport of fatty acids through the mitochondrial membrane (and therefore ATP synthesis), improves oocyte developmental competence in the mouse [225,226]. Indeed, the optimal balance of fatty acid oxidation during oocyte maturation seems to be a way to enhance the quality of the produced embryo [202].

### 4.3. Comparative Dependence to Lipid Metabolism between Mammal Species

**Figure 4:**
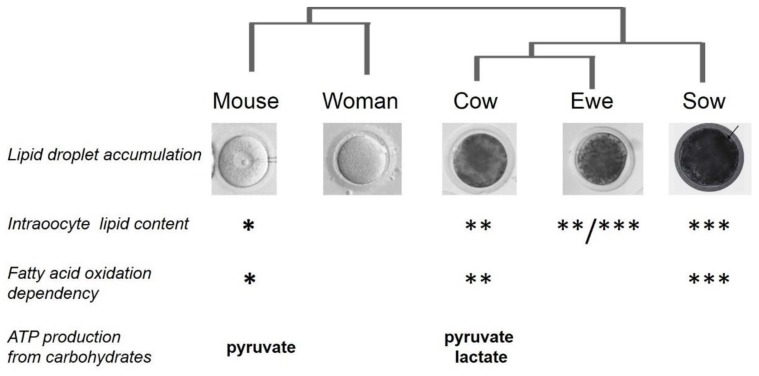
Importance of carbohydrate and lipid metabolism during oocyte maturation in five mammalian species. Brightfield microscopy pictures show the darkening of the oocyte cytoplasm (from mouse to sow) reflecting the increasing content in lipid droplets, coherent with the fatty acid amount from triglycerides and phospholipids [228,229], and the dependency on fatty acid oxidation, as assessed by the impact of pharmacological inhibition of carnitine palmitoyltransferase 1 [201,203]. The number of asterisks correlates with the amount/importance of these parameters. Carbohydrate metabolism is activated from distinct substrates.

Even if the amount of oocyte lipids is much lower in mammals than in birds and fish, since in the latter two, the egg lipids are necessary for the growth of fetuses and larvae, we will focus here on comparisons between mammalian oocytes. Analyses of lipid profiles enabled us to discriminate different ovarian compartments, as mentioned above, but they also made it possible to compare species. Indeed, lipid analyses, performed with MALDI-TOF MS, notably based on comparisons of the relative abundance of major glycerophospholipids, sphingolipids and glycerolipids, are capable of discriminating the oocytes and embryos of various species (human, bovine, ovine, fish and insect) [227]. Differences in lipid composition between species suggest differences in their lipid metabolism at the gonad level. These observations on lipid profiles are confirmed by measures of total lipid amounts performed on oocytes from different species. Oocyte lipid content shows great disparity between differing mammalian species, as reflected by their light or dark appearance (Figure 4). Oocytes of dogs and pigs contain the highest amount of lipids (161 ng in the pig), with a higher number of lipid droplets [228]. Oocytes of cows and ewes also contain a high albeit lower level of lipids (63 ng and 89 ng respectively), and they exhibit fewer and smaller lipids droplets. Finally, oocytes from mice and humans contain the lowest amount of lipids [194]. The abundance of neutral lipids in lipid droplets correlates with dark inclusions in the ooplasm, and was quantified by lipid-specific fluorescence [202,229]. The expression of carnitine palmitoyltransferase 1, which initiates mitochondrial oxidation of long-chain fatty acids, correlates with lipid abundance in these species [201,223] (Figure 4). Among the 24 fatty acids detected in porcine, bovine and ovine oocytes, the 3 most abundant are palmitic acid (16:0; 25–35%, *m*/*m*), stearic acid (18:0; 14–16%), and oleic acid (18:1n-9; 22–26%). Saturated fatty acids represent, on average, 45–55% of total fatty acids (m/m) in oocytes, and are more abundant than monounsaturated fatty acids (MUFA: 27–34%) or PUFA (11–21%).

Among n-6 PUFA, linoleic acid (18:2n-6; 5–8%, *m*/*m*) and arachidonic acid (20:4n-6; 1–3%) are the most abundant fatty acids. Phospholipids represent 25% of lipids in these species. Concerning fatty acids trapped in the triacylglycerol fraction, the porcine oocyte contains 74 ng, compared to 23–25 ng in ruminant oocytes. These differences in oocyte lipid composition between species could be related to differences in embryo sensitivity to drop in temperature, culture and cryopreservation [228]. As the lipid profile of the oocyte could be an indicator of future embryo quality or of its tolerance to cryopreservation, the supplementation of embryo donor cows is considered as a tool to modulate oocyte lipid content and, therefore, the quality of the resulting embryo. Indeed, conjugated linoleic acid supplementation, when compared to stearic acid supplementation, is able to modulate the lipid composition of plasma and follicular fluid, but also of the oocyte after in vitro maturation [230,231].

Moreover, the importance of fatty acid oxidation varies between species [201]. Indeed, the requirement for lipid metabolism during maturation seems to be correlated with the total amount of fatty acids in the oocyte. In species where the oocyte contains a moderate amount of lipids, as in mice and humans, carbohydrate metabolism (through pyruvate) is the main energy source, and it is capable of compensating for the inhibition of fatty acid oxidation to meet energy requirements. In contrast, in species where the oocyte contains a high level of lipids, as in swine, the dependency on lipid metabolism seems to be high, and even a partial inhibition of fatty acid β-oxidation is sufficient to block oocyte maturation. Mouse oocytes should be less sensitive to fatty acid β-oxidation impairment than swine oocytes, with an intermediate status for human, cows and ewes [194]. The oocyte capacity for lipid storage may also vary between species. Indeed, the expression level of diacylglycerol *O*-acyltransferase 1 (DGAT1), and therefore, the rate of TAG synthesis and storage vary between species, and are the highest in the swine oocyte. The cause of such variations between species is not known at the moment, but it is thought to be related to the extended duration of embryo development before implantation, and to the litter size in high lipid containing pig and dog oocytes [194]. However, this correlation between time to implantation and lipid content is correct for extreme species (high or low lipid content), but not with intermediate species (cows, ewes, goats). The explanation dealing with litter size is also not correct when comparing pig with mouse, which also produce sizeable litters while their oocytes contain low amount of lipids.

To conclude on oocyte lipid metabolism, huge differences exist between mammalian species. These differences are due to overall metabolic orientation between monogastric versus ruminant species. They are also due to a variable dependency onto lipid metabolism. Indeed, rodents and human oocytes rely mostly on glucose and pyruvate metabolism, whereas ruminant and pig oocytes rely more on lipid metabolism. Lipid metabolism is still poorly understood, and the relationships between lipid and fatty acid composition remain to be explored in the oocyte. Moreover, the relationship between lipids/fatty acids and modulations of oocyte functions remain unknown, meaning that their mechanisms of action need to be further investigated. Some data exist on specific fatty acids in a given species, but work is needed to have a better understanding of the lipid action in the oocyte.

## 5. Conclusions

This comparative analysis of some aspects of oocyte biology reflects that the factors responsible for differences between species are diverse. They likely involve the fact that different sets of genes are expressed by oocytes of different species, because some genes were lost in some but not all species, such as *SLBP2, BCAR4,* some *ZP*, and/or because distinct paralogues are selectively activated. A second point is linked to the relative difference in the respective roles of *GDF9* and *BMP15* as motors of follicular growth in mammals, and the relative importance of these two factors in regulating the number of ovulations. Finally, the variable importance of lipid metabolism in the oocyte undoubtedly plays an important role in the differences in the management of interactions between metabolism and reproduction among mammalian species. Overall, these differences remind us that the molecular mechanisms underlying oocyte biology should be extrapolated from one species to another with extreme caution, and highlight the importance of carrying out experimental investigations on a variety of mammalian animal models.
